# Mercury Contamination: A Growing Threat to Riverine and Urban Communities in the Brazilian Amazon

**DOI:** 10.3390/ijerph19052816

**Published:** 2022-02-28

**Authors:** Heloisa do Nascimento de Moura Meneses, Marcelo Oliveira-da-Costa, Paulo Cesar Basta, Cristiano Gonçalves Morais, Romulo Jorge Batista Pereira, Suelen Maria Santos de Souza, Sandra de Souza Hacon

**Affiliations:** 1 Programa de Pós-Graduação em Ciências da Saúde (PPGCSA), Universidade Federal do Oeste do Pará, Rua Vera Paz Av. Vera Paz, s/n, Bairro Salé, 1° Pavimento, Unidade Tapajós, 68035-110 Santarém, Brazil; cristiano.goncalves.moraes@gmail.com (C.G.M.); romulo.jorge55@gmail.com (R.J.B.P.); 2 WWF-Brasil, CLS 114 Bloco D-35, 70377-540 Brasília, Brazil; marcelo@wwf.org.br; 3 Escola Nacional de Saúde Pública Sérgio Arouca, Fundação Oswaldo Cruz, Rua Leopoldo Bulhões, 1480, Manguinhos, 21041-210 Rio de Janeiro, Brazil; paulobasta@gmail.com (P.C.B.); shacon@ensp.fiocruz.br (S.d.S.H.); 4 Programa de Pós-Graduação em Sociedade Natureza e Desenvolvimento (PPGSND), Universidade Federal do Oeste do Pará, Rua Vera Paz Av. Vera Paz, s/n, Bairro Salé, 1° Pavimento, Unidade Tapajós, 68035-110 Santarém, Brazil; s.s.souza@hotmail.com

**Keywords:** gold mining, blood sampling, Tapajós basin, biochemical markers, Santarém

## Abstract

In recent decades, widespread and uncontrolled use of mercury (Hg) in artisanal small-scale gold mining has released thousands of tons of mercury-contaminated waste in the Amazon biome, endangering the largest tropical rainforest worldwide. In this study, we assessed and compared blood Hg levels in individuals living in urban and riverine areas in the lower Tapajós basin and examined the association between Hg exposure and specific biochemical parameters. In total, 462 adults from eight riverine communities and one urban area were assessed. Overall, 75.6% of the participants exhibited Hg concentrations exceeding the safe limit (10 µg/L). Hg exposure was higher in the riverine population (90%) than in urban areas (57.1%). Mean Hg levels were 21.8 ± 30.9 µg/L and 50.6 µg/L in urban and riverine residents, respectively. The mean Hg level was higher in those aged 41–60 years in both urban and riparian areas, with riparian residents exhibiting a mean double that of urban residents. The highest glucose and hepatic biomarker levels were detected in the urban area, whereas the highest levels of renal biomarker occurred in the riverine population. Our results indicate that Hg contamination remains a persistent challenge for the urban population of Santarém, a major city in the Brazilian Amazon.

## 1. Introduction

Mercury (Hg) is one of the most harmful chemicals on earth and represents a global public health challenge. Although a naturally occurring element, human activities have increased the total atmospheric Hg concentration by approximately 450% above natural levels [1]. In Latin America, artisanal small-scale gold mining (ASGM) remains the primary source of Hg emissions and contamination [2], and along with biomass burning and deforestation, accounts for most of the Hg released in the Amazon [3].

Hg has been used to extract gold for centuries and is still a widely employed technique in Southern America because it is an easy and inexpensive process. Over the last four decades, the widespread, unregulated, and uncontrolled use of Hg in ASGM has released thousands of tons of mercury-contaminated waste in the Amazon biome. In the Brazilian Amazon, ASGM was found to be responsible for environmental contamination, as well as wildlife and human exposure over the years [4]; however, the magnitude of exposure remains unclear owing to the illegality of the sector, hampering credible data on the amount of Hg released in the environment [5]. For individuals living in the Amazon, fish consumption is the main source of exposure [6,7], which endangers the food security and livelihood of traditional communities in the region [8].

Hg exposure may cause various important health issues, including damage to the neurological, cardiovascular, immunological, and digestive systems, lungs, kidneys, skin, and eyes [9,10,11]. Organic forms of Hg, such as methylmercury (MeHg), are particularly toxic to humans and are rapidly absorbed by the body, widely distributed in all tissues, and slowly eliminated [3]. Maternal Hg exposure has been associated with a series of developmental effects, and it may cause several chronic lifelong disorders [12,13]. Such heterogeneous toxicity translates into a complex variety of possible clinical manifestations induced by mercury intoxication, complicating clinical diagnostic and epidemiological studies [14,15].

The Tapajós basin is the fifth largest tributary of the Amazon basin, covering approximately 492,000 km^2^ and plays important social, cultural, and economic roles. However, instead of promoting socially inclusive and environmentally sustainable development in the region, federal administrations have supported controversial activities, including gold mining. This includes the creation of the gold mining zone, Reserva Garimpeira do Tapajós, in 1983, which attracted thousands of people towards the search for gold.

High gold prices in the international market and political instability have boosted illegal gold mining in the Brazilian Amazon. The rate of deforestation due to illegal mining increased by more than 90% from 2017 to 2020, reaching 101.7 km^2^ in 2020 [16]. Furthermore, there is a disconnection between the Brazilian health information system and cases of Hg contamination in the Amazon [17], with a critical deficiency in the information system regarding the data on human Hg intoxication from the Amazon States. This is of great concern, as it undermines the ability of decision-makers and government agencies to develop strategies and actions to mitigate this issue. Since 1980, several studies assessing human Hg exposure in the Brazilian Amazon were conducted in the Tapajós basin [4,18,19,20]. Although these studies reported widespread Hg contamination in the basin, few investigated the urban population. This is probably due to the prioritization of riverine and indigenous populations, which are among the most vulnerable communities in the Amazon, with scarce access to the healthcare system and high dependency on fish as their main protein source.

The objectives of the present study were to (1) evaluate and (2) compare Hg levels in blood samples from residents of urban and riverine areas in the lower Tapajós basin. Our study also (3) describes the health situation of urban and riverine residents in the lower Tapajós River. We aim to contribute to qualifying the magnitude of Hg contamination in the Amazon and support the development of national and subnational strategies to manage this issue, as well as present evidence indicating the threat to human well-being in the region.

## 2. Methods

### 2.1. Study Design and Population

**Figure 1:**
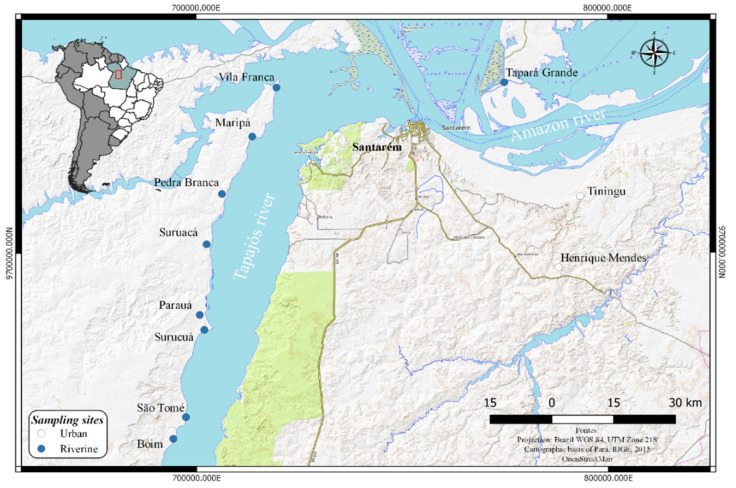
Map of the study area indicating sampling sites.

A cross-sectional investigation was conducted between 2015 and 2019. Participants from riverine communities and one urban area along the lower Tapajós basin were voluntarily recruited with the support of local community leaders, followed by meetings in the communities. A total of 462 adults (>18 years old) from eight communities (Vila Franca, Maripá, Pedra Branca, Suruacá, Parauá, Surucuá, São Tomé, and Boim) and one community from the Amazon River (Tapará Grande) and the urban area of Santarém (Figure 1) were selected, excluding pregnant women and people living in indigenous communities, mostly because these groups were the main target in previous studies. Sociodemographic data, gender, age, frequency of fish intake, and educational level (years of schooling) were obtained through an interview-administered questionnaire adapted from protocols for environmental and health assessments. Based on questionnaires, no subject of this study had an established record of involvement with ASGM. Questionnaires with inconsistencies in terms of some variables, notably due to recording failures, were discarded. At the end of the interview, all participants were weighed and measured, body mass index (BMI = weight [kg]/height [m]^2^) was calculated, and blood pressure was monitored. The participants were categorized into illiterate, elementary education (up to 12 years of study), or higher education (over 12 years of study) groups. Fish consumption was classified as follows: daily consumption, regular consumption (1 to 3 times a week), and occasional consumption (maximum 2 times a month).

All procedures were approved by the Ethics Committee of Pará State University (UEPA) (Technical Report No. 1,127,108), and all participants provided written informed consent prior to the interviews.

### 2.2. Blood Sampling and Analysis

We collected 10 mL of venous blood by venipuncture from each participant, which were then divided into two samples: 5 mL were added to a tube with EDTA anticoagulant for Hg analysis, and the remaining 5 mL were poured into a tube without anticoagulant to measure serum levels of renal and hepatic markers. All tubes were stored in thermal boxes for transportation to the laboratory. Tubes containing anticoagulants were stored in a freezer at a temperature below 0 °C until dosage. The sample tubes without anticoagulant were centrifuged at 3500 rpm for 5 min, and the clot was discarded using a micropipette, which is crucial because hepatic enzymes in contact with blood cells undergo degradation, which may mask hepatic alterations. The serum amount was measured on the sampling day and the remaining serum was stored in a freezer for replicate measurements, if necessary. Sampling procedures, storage, and transportation were performed in accordance with the guidelines recommended by the Brazilian Society of Clinical Analysis.

Total Hg concentrations in the blood were analyzed by atomic absorption spectrometry using a direct mercury analyzer (DMA-80; Milestone Inc., Santa Clara, CA, USA). The accuracy of the results was determined using a calibration curve with certified reference materials (CRM) (Seronorm™ Trace Elements Whole Blood, Billingstad, Norway). The detection limit of the assay was 0.2 µg/L, and samples were analyzed in duplicate. Hg exposure level was defined as low exposure (≤10 µg/L) or high exposure (≥10 µg/L), following the threshold set for blood (10 μg/L) by the World Health Organization (WHO) [21]. Concentrations of biochemical markers were determined, including glucose (non-fasting), urea, and creatinine (for kidney function), alanine aminotransferase (AST), and aspartate aminotransferase (ALT) (for liver function). The analyses were performed using the BS 200 equipment from MINDRAY, according to the manufacturer’s instructions. Creatinine and urea values are expressed in milligrams per deciliter (mg/dL), and AST and ALT results are expressed in units per liter (U/L); the reference values adopted varied according to the participant’s age. All biochemical markers were analyzed in a certified laboratory and “LabTest Diagnóstica” kits were used. The CRM varies according to the type of analysis performed, being: Enzymatic Glucose Oxidase-Peroxidase (reference value 133—glucose), Kinetic UV Urease (GLDH) (reference value 104—urea), Enzymatic Colorimetric (reference value 96—creatinine), and Kinetic UV-IFCC Without Pyridixal Phosphate (reference value 109—AST and reference value 108—ALT).

### 2.3. Statistical Analysis

Descriptive statistics (mean, median, standard deviation) were used to examine Hg levels and biomarker concentrations in relation to the participant’s residence location (urban or riverine). The epidemiological profile was evaluated according to Hg exposure level (low or high) and participants’ residence location (urban or riverine) using Pearson’s Chi-square test for heterogeneity. In addition to descriptive analyses, potential associations considering Hg levels and (1) age, (2) gender, (3) schooling, and (4) fish consumption were evaluated using the Shapiro-Wilk test to verify normally distributed data, and the Kruskal–Wallis test was applied as the non-parametric method, and the Spearman’s test was used for the age correlation analysis. A health situational diagnosis analysis of the research participants was conducted, detailing the occurrence of altered biochemical parameters, blood pressure, and BMI in urban and riverine residents.

All statistical tests were performed using STATA software version 16, with a significance level of 5%.

## 3. Results

Hg levels were detected in all 203 individuals from Santarém City and 259 participants from riverine communities assessed in the present study. Among the participants, 75.6% exhibited Hg levels exceeding the safe limit set by the WHO and were defined as the high-exposure group. Mean Hg levels in the high exposure group were 48.3 ± 58.5 µg/L, whereas the low exposure group showed a level of 6.2 ± 2.2 µg/L. Among the 349 participants in this group, 77.1% had Hg levels ranging between 10 and 50 µg/L, 11.2% between 51 and 100 µg/L, 6.9% between 101 and 200 µg/L, and 4.9% exhibited Hg levels >200 µg/L. Strikingly, the highest Hg level (296.5 µg/mL) was detected in a 47-year-old woman living at a riverine site, almost 30-fold higher than the safe limit.

The prevalence of Hg exposure was higher in the riverine population (90%) than in those living in urban areas (57,1%), with statistically significant differences between the two categories (χ^2^ = 63.3; *p* < 0.0001). Mean Hg levels in urban area residents were 21.8 ± 30.9 µg/L, with levels of 33.6 µg/L Hg and 5.9 µg/L detected in the high and low exposure groups, respectively. The highest Hg level in an urban area resident was 247.1 µg/L. In the riverine participants, the mean Hg level was 50.6 µg/L, and the high exposure group exhibited a level of 55.5 µg/L Hg; the highest Hg level noted was 296.5 µg/L.

Regarding participants’ age, 42.1% were aged between 41–60 years old, and 39.5% were 21–40 years old. In this older group (41–60), the mean Hg level was 44.3 ± 59.3 µg/L, whereas the younger group (21–40) showed Hg levels of 32.1 ± 49.6 µg/L (Table 1). Considering gender, 65.6% were female with a mean Hg level of 32.7 ± 47.3µg/L, (minimum = 1.4 µg/L and maximum = 296.5 µg/L), and 34.4% were males displaying a mean Hg level of 48.1 ± 63.9 µg/L (minimum = 1.7 µg/L and maximum = 288.3 µg/L). In terms of schooling, 73.1% of participants were in the elementary education category; however, the highest level of Hg was detected in the illiterate group (45.8 ± 50.9 µg/L), while 24.3% of participants (higher education category) showed the lowest Hg level (17.3 ± 31.6 µg/L). Regarding fish consumption, most participants (*n* = 235) reported regular consumption. Unsurprisingly, the group with daily fish consumption habits showed the highest Hg level (48.1 ± 59.9 µg/L).

The prevalence of Hg exposure in the riverine participants along the Tapajós River (59.5%) was higher than that along the Amazon River (40.5%).

The profile was also described in relation to the participant’s location of residence (Table 2). In the urban area, the mean Hg level was 33.6 ± 36.7 µg/L, while a level of 55.5 ± 65.7 µg/L was recorded in the riverine area. The prevalence of high exposure was greater among riverine residents (PR = 1.57, 95% confidence interval [CI] = 1.38–1.78, *p* = 0.000), with a statistically significant difference when comparing median Hg levels among the two residence locations (χ^2^ = 78.684, *p* = 0.0001). Moreover, differences were observed when comparing urban and riverine areas of the Amazon River (χ^2^ = 6.46, *p* < 0.0001) and between urban and riverine areas of the Tapajós River (χ^2^ = 8.13, *p* < 0.0001), but not between participants in the two riverine areas (χ^2^ = −0.72, *p* = 0.7009).

The mean Hg level was higher in participants aged 41 to 60 years in both urban and riparian areas (28.7 ± 37.3 µg/L and 55.6 ± 68.8 µg/L, respectively), with the riparian area recording a mean nearly two-fold that of the urban area. A statistically significant difference was observed among all age groups when comparing urban and riparian areas.

Although both areas showed a higher number of females (64% in the riverine and 67% in the urban area) at a higher exposure risk, Hg levels were higher among males in both riverine and urban areas (63.4 ± 70.7 µg/L and 26.5 ± 46.0 µg/L respectively). For both sexes, a higher Hg level was observed in the riverine area than that in the urban area (χ^2^ = 36.05; *p* = 0.0001 female; χ^2^ = 44.02; *p* = 0.0001 male).

Regarding schooling, most participants of both locations were categorized into elementary education group, but of 111 individuals in the higher education category, 89 were from the urban area exhibiting the lowest mean Hg level (12.2 ± 12.8 µg/L). In riverine areas, participants from both elementary and higher education groups showed higher levels of Hg (χ^2^ = 28.16; *p* = 0.0001 and χ^2^ = 15.08; *p* = 0.0001, respectively).

Regarding the frequency of fish consumption, the categories “daily” and “ regular” were, as expected, the most reported in urban and riverine areas. We detected a statistically significant difference between urban and riparian areas in all fish consumption categories.

For the total samples (with no epidemiological categories), we evaluated four potential associations: (1) Hg level and age, demonstrating a positive and statistically significant correlation (r_s_ = 0.23; *p* < 0.0001). A similar correlation was observed for the urban area (r_s_ = 0.37; *p* < 0.0001), whereas in riverine areas, this correlation was not statistically significant (r_s_ = 0.12; *p* = 0.06). (2) Hg level and sex: men had higher Hg levels than women (χ^2^ = 9.10, *p* = 0.0025). In females, a positive and statistically significant correlation was detected between age and Hg levels (r_s_ = 0.26; *p* < 0.0001), but no significant correlation was observed between age and Hg levels in male participants (r_s_ = 0.11; *p* = 0.16). (3) The Hg level and schooling resulted in a statistically significant difference (χ^2^ = 67.84, *p* = 0.0001), with a statistically significant difference between elementary and higher education (8.15, *p* < 0.0001), as well as between illiterate and higher education (χ^2^ = 3.31, *p* = 0.0014). (4) The Hg level and fish consumption were significantly different (χ^2^ = 88.62, *p* = 0.0001), with a higher difference between daily and occasional fish consumption (χ^2^ = 9.37, *p* < 0.0001).

The situational health diagnosis conducted in all participants showed that the median biomarker levels were within the reference range. The highest glucose and hepatic biomarker levels were documented in urban area residents; the highest levels of renal biomarkers were noted in participants living in the riverine area (Table 3).

Regarding renal markers, it was observed that 19 residents presented altered levels of urea, of which 16 (84.2%) were from the riverine area, 3 (15.8%) from the urban area, and 6 residents presented altered levels of creatinine, of which 5 (83.3%) were from the riverine area (Table 4). The highest levels of urea (115.7 mg/dL) and creatinine (4.4 mg/dL) were observed in the same participant, a 65-year-old woman from the riverine area, who presented a Hg level of 122.8 µg/L.

For liver markers, 25 participants (88.0% from urban area) presented AST levels above the reference value, while 18 participants (94.4% from urban area) showed altered TGP levels. A 44-year-old woman (Hg level = 50.2 µg/L) and a 53-year-old woman (Hg level = 42.4 µg/L), both from urban area, presented elevated levels of AST and ALT: 101.0 U/L of AST and 143.0 U/L of ALT and 232.0 U/L of AST and 120.0 U/L of ALT, respectively.

Glucose, BMI, and blood pressure levels were measured in 191 participants: 98 from the riverine area and 93 from the urban area. 24 of the participants (12.6%) had altered glucose levels, of which 15 (62.5%) had Hg levels above 10 µg/L. Blood pressure was altered in 28 patients (14.7%), of which 23 (82.1%) were exposed to elevated levels of Hg. In relation to BMI, 113 participants presented altered weight, of which 63 were overweight and 50 had some degree of obesity, of which 90 (79.6%) presented Hg levels above the mercury threshold.

## 4. Discussion

Few studies have addressed Hg exposure in individuals living in urban areas of the Amazon. Most research projects have focused on riverine [22] and indigenous people [7]. Our findings indicate that Hg contamination remains a threat to riverine communities and impacts the urban population in the Tapajós basin. In addition, all participants included in the present study had high Hg levels; more than three-fourths of the participants exhibited levels exceeding the safety limit set by the WHO, representing a chronic public health issue in the Tapajós region as advocated in previous studies [18,22,23,24,25].

Our study differs from other studies performed in the Tapajós Basin, with respect to mainly evaluating residents of the urban area of Santarém and riverside communities that have not been studied yet, as well as a community from the Amazon River. Previous studies were conducted mainly in the Middle Tapajós region, whereas ours was conducted in the Lower Tapajós. To our knowledge, four studies have targeted the population of Santarém, but using different biomarkers of exposure, and consequently undermining correlation between results: (1) in 1995, hair samples from 10 individuals were evaluated and the authors found a mean Hg level of 2.7 μg/g [26]; (2) in 2000, urine from 50 urban workers were sampled and the mean Hg level found was 57.5 µg/L [27]; (3) in 2003, hair samples from 44 individuals were evaluated and the average Hg level found was 3.1 μg/g among residents of the Mapiri neighborhood and 0.8 μg/g among university students [28]; and (4) in 2010, hair samples from 70 individuals were evaluated, resulting in an average Hg level of 1.5 μg/g in women and 2.52 μg/g in men [29].

The high exposure group presented rates nearly 4-fold higher than the safety limit, reaching almost 30-fold in one analyzed participant. Although the urban population has also been exposed, riverine populations remain the most vulnerable to Hg exposure, presenting a higher proportion of Hg contamination. This can be explained by evaluating dietary habits [8], as fish remains the predominant protein consumed by riverine populations, while individuals living in cities have access to a greater variety of food [30]. Accordingly, accumulated evidence could trigger additional alerts related to food security. Among these fish-dependent populations, the prevalence of Hg exposure was higher in the riverine population (90%) than in those living in urban areas (57.1%), thus complicating the proposition of mitigating measures.

Along with the regional dietary patterns, the Hg bioaccumulation feature may also explain the higher Hg levels found in older males. The 40–60-year-old group exhibited relatively higher Hg levels than the 21–40-year-old participants, with males presenting higher levels than females. As Hg contamination is biocumulative [31] and males usually consume larger meals, it is reasonable to assume that over the years, frequent or daily fish consumption by males, particularly among fishermen families [32], may have led to greater Hg levels in older males.

Among the total sampled females, 64.7% were of childbearing age (18 to 49 years), and of these, 69.9% had mean Hg levels of 36.2 µg/L. This raises serious concerns, given the higher risk of congenital malformations in newborns whose mothers were exposed to Hg during pregnancy [33,34]. MeHg reportedly crosses the placental and blood-brain barriers and causes more damage to the developing central nervous system than the adult central nervous system. According to Bertossi et al. [35], small amounts of MeHg are sufficient to induce developmental problems in the fetus. Therefore, it is of paramount importance to monitor Hg levels in women of childbearing age, pregnant women, and their newborns to prevent the effects of mercurial exposure on infants.

Notably, Hg compounds in the blood reflect recent and current exposure [36]. Accordingly, considering that sampling occurred between 2015 and 2019, it might be reasonable to assume that our results fail to reflect the current scenario in the Tapajós basin, which has reportedly worsened over the last two years, according to several researchers and press. Given the explosion of new gold mining sites in the Munduruku Indigenous territory and other protected areas in the Tapajós basin over the past two years [37], there has been an increase in the indiscriminate use of Hg upstream of the study area. Furthermore, the economic and social crisis triggered by the coronavirus (COVID-19) pandemic has pushed local communities to look for cheaper sources of animal protein. As part of the culture of riverbank populations, fishing efforts have increased, as have the consumption of fish with elevated levels of Hg [38].

Moreover, in a hypothetical scenario, it is critical to estimate the extent of the issue if the data obtained from this study were extrapolated to the urban population of Santarém, approximately 200,000 inhabitants, which would result in nearly 114,000 people at risk of high levels of Hg contamination. This absolute number must be questioned, as it depends on important variables such as diet, sex, sample representativeness, frequency of fish consumption, age, exposure to other sources of Hg, such as atmospheric contamination, either by burning emissions or Hg burning [39,40] in gold processing centers, along with other confounding variables. However, even if the total number is overestimated, it is reasonable to assume that human health effects following Hg contamination, such as mental health impairments [41], as well as behavioral, immunological, hormonal, and reproductive changes [42], among others, can be a reality in the region, representing additional expenses for the local public healthcare system. Alternatively, considering the limited healthcare in the region, this might result in an immeasurable loss in the quality of life for thousands of people.

Although Hg is a toxic heavy metal associated with tissue damage and can cause different diseases, few studies have examined the effects of Hg exposure on liver and kidney functions [43]. The health situational analysis showed that both urban and riverside area residents presented alterations in the evaluated health indicators. Alterations in kidney markers were more frequent in riverine residents, and alterations in liver markers were more frequent in urban area residents. Although these are not the most frequently used markers for making an association between Hg and kidney or liver damage, it is notable that the highest levels of these markers were recorded in people with high Hg levels.

Regarding blood pressure, participants with hypertension in both urban and riverine areas were observed. Hu et al. [44] conducted a systematic review and meta-analysis to clarify the association between hair Hg levels and hypertension. On reviewing 29 studies, the authors revealed that the pooled OR for hypertension, comparing the highest and lowest Hg exposure categories, was 1.35 (95% CI: 0.99, 1.83) for populations with hair Hg ≥ 2 µg/g compared with the OR of 1.12 (95% CI: 0.82, 1.52) for populations with hair Hg < 2 µg/g. These findings suggest a significant positive association between hair Hg levels and hypertension. Considering that 85.7% of people with hypertension also presented high levels of mercury, it is reasonable to consider an association between high levels of Hg and hypertension in the population studied.

Despite these findings, some limitations must be considered. First, the campaigns for data collection occurred during an extended time-lapse, covering 2015 to 2019, making comparisons susceptible to information bias. Another limitation is related to the possibility of comparing results, as most studies in the Amazon have examined urine [45] or hair [22] to study biomarkers of Hg exposure. In addition, the representativeness of the overall population could be impaired by the voluntary recruitment of participants. Finally, a broad investigation to clarify dietary differences between riverside and urban communities is needed, as there are relevant differences between fish consumption in urban and rural communities and between Brazilian geographic regions [6]. In summary, our findings should be interpreted with caution and considered exploratory.

## 5. Conclusions

People living in communities in the Amazon have been exposed to Hg for decades through the ingestion of mercury-contaminated fish. Using a biomarker of recent exposure, our results show that Hg levels are high among the participants. All the individuals analyzed, both from urban and riverine areas, who reported frequent consume of local fish, had detected Hg levels and were consequently exposed to negative health effects. High levels of this metal were found in people of all age groups, both genders, and all levels of education. However, the highest levels were registered in people who reported high daily fish consumption. This indicates that mercury exposure is not only linked to the residence location, but directly influenced by contaminated fish consumption.

To our knowledge, Santarém is far from any goldmining sites. Nevertheless, the local people assessed are exposed to different levels of Hg. Thus, we conclude that exposure to mercury is not restricted to the goldmining site areas but can occur throughout much of the river basin that is greatly impacted by the goldmining activity. We advocate the development of more comprehensive studies to identify mercury contamination from sources other than fish, such as atmospheric and deforestation Hg emissions.

It is critical to assess the impacts of Hg contamination on local communities and their environment and build scientific evidence that helps to develop effective mitigation measures and policies. Therefore, tackling the health, environmental and social impacts from Hg contamination in the region should be among the main goals for governments and decision-makers. Health surveillance programs tailored in accordance with the social and environmental heterogeneity in the Amazon could be the turning point in this issue, claimed for decades as a dangerous threat to the human population.

## Figures and Tables

**Table 1:** Epidemiological characteristics of participants according to Hg levels, Brazilian Amazon basin, 2015–2019.

Variables	Low Exposure		High Exposure		Total Hg Level in the Blood (μg/L)					
	*n*	%	*n*	%	Mean	Median	Standard Deviation	Min	Max	Kruskal-Wallis
Age range (*n* = 451) *										
18–20 (*n* = 21)	10	47.6	11	52.4	20.9	12.0	27.6	3.7	129.6	χ ^2^ = 19.05 *p* = 0.0003
21–40 (*n* = 178)	54	30.3	124	69.7	32.1	15.0	49.6	1.4	288.3	
41–60 (*n* = 190)	37	19.5	153	80.5	44.3	23.5	59.3	1.6	296.5	
>61 (*n* = 62)	10	16.1	52	83.9	39.7	22.1	49.6	3.3	271.9	
Gender (*n* = 462)										
Female (*n* = 303)	80	26.4	223	73.6	32.7	16.8	47.3	1.4	296.5	χ^2^ = 9.11 *p* = 0.003
Male (*n* = 159)	33	20.8	126	79.2	48.1	25.5	63.9	1.7	288.3	
Schooling (*n* = 457) *										
Illiterate (*n* = 12)	3	25.0	9	75.0	45.8	30.7	50.9	7.3	185.5	χ^2^ = 67.85 *p* = 0.0001
Elementary education (*n* = 334)	52	15.6	282	84.4	44.7	24.0	58.5	1.4	296.5	
Higher education (*n* = 111)	57	51.4	54	48.6	17.3	9.6	31.6	1.6	296.5	
Fish consumption (*n* = 457) *										
Daily (*n* = 146)	5	3.4	141	96.6	48.1	26.1	59.9	7.0	296.5	χ^2^ = 88.62 *p* = 0.0001
Regular (*n* = 235)	57	24.3	178	75.7	40.7	20.1	56.1	1.4	296.5	
Occasional (*n* = 76)	47	61.8	29	38.2	12.1	7.6	14.2	1.6	80.1	
Home location (*n* = 462) *										
Riverine—Amazon River (*n* = 105)	16	15.2	89	84.7	45.3	27.3	55.2	3.3	271.9	χ^2^ = 79.21 *p* = 0.0001
Riverine—Tapajós River (*n* = 154)	10	6.5	144	93.5	54.3	25.0	69.3	1.4	296.5	
Urban (*n* = 203)	87	42.9	116	57.1	21.8	11.0	30.9	1.7	247.1	

* χ^2^ of person: Comparison of variables such as age, schooling, fish consumption, and location of residence as a function of mercury exposure (high exposure × low exposure) shows statistically significant results.

**Table 2:** Epidemiological profiling of participants according to residence location, Brazilian Amazon basin, 2015–2019.

Variables	Residence Location	*n*	%	Total Hg Level in the Blood (μg/L)					
				Mean	Median	Standard Deviation	Min	Max	Kruskal-Wallis
Age range									
18–20 (*n* = 21) *	Riverine	12	57.1	30.4	19.9	33.6	6.9	129.6	χ^2^ = 9.78 *p* = 0.0019
	Urban	9	42.9	8.2	6.6	6.1	3.7	23.8	
21–40 (*n* = 178) *	Riverine	98	55.1	45.7	25.0	58.7	1.4	288.3	χ^2^ = 59.19 *p* = 0.0001
	Urban	80	44.9	15.4	8.5	27.9	2.3	222.7	
41–60 (*n* = 191) *	Riverine	112	58.6	55.6	28.6	68.8	1.6	296.5	χ^2^ = 15.92 *p* = 0.0001
	Urban	79	41.4	28.7	14.9	37.3	1.7	247.1	
>61 (*n* = 62) *	Riverine	31	50.0	53.8	27.0	64.9	5.8	271.9	χ^2^ = 5.13 *p* = 0.0235
	Urban	31	50.0	24.7	17.5	21.1	3.3	80.1	
Gender									
Female (*n* = 303) *	Riverine	166	54.8	43.6	21.6	59.1	1.4	296.5	χ^2^ = 36.05 *p* = 0.0001
	Urban	137	45.2	19.5	10.1	20.0	2.3	102.4	
Male (*n* = 159) *	Riverine	93	58.5	63.3	35.0	70.4	3.3	288.3	χ^2^ = 44.02 *p* = 0.0001
	Urban	66	41.5	26.5	11.2	46.0	1.7	247.1	
Schooling									
Illiterate (*n* = 12)	Riverine	7	58.4	50.9	14.8	66.8	7.3	185.5	χ^2^ = 0.53 *p* = 0.4649
	Urban	5	41.7	38.6	34.8	18.1	21.1	67.3	
Elementary education (*n* = 334) *	Riverine	226	67.7	52.2	27.3	64.5	1.4	296.5	χ^2^ = 28.16 *p* = 0.0001
	Urban	108	32.4	28.9	15.4	39.0	1.7	247.1	
Higher education (*n* = 111) *	Riverine	22	19.8	37.8	18.5	63.2	1.6	296.5	χ^2^ = 15.08 *p* = 0.0001
	Urban	89	80.2	12.2	8.7	12.8	2.3	82.8	
Fish consumption									
Daily (*n* = 146) *	Riverine	128	87.7	50.0	27.3	60.6	6.7	296.5	χ^2^ = 3.731 *p* = 0.0534
	Urban	18	12.3	34.8	21.6	54.6	8.4	247.1	
Regular (*n* = 235) *	Riverine	123	47.7	52.9	25.4	68.9	1.4	296.5	χ^2^ = 14.03 *p* = 0.0002
	Urban	112	52.3	27.2	14.6	32.5	1.7	222.7	
Occasional (*n* = 76) *	Riverine	7	9.2	28.6	16.6	24.3	1.6	74.9	χ^2^ = 6.831 *p* = 0.0090
	Urban	69	90.8	10.4	6.7	11.8	2.4	80.1	

* Kruskal–Wallis: Comparison of residence location (urban × riverside) showed a statistically significant result.

**Table 3:** Descriptive analyses of the biochemical marker concentrations in participants’ blood samples, according to residence location (riverine vs. urban), Brazilian Amazon basin, 2015–2019.

Biochemical Markers	Reference Values	Residence Location	*n*	Blood levels				
				Mean	Median	Standard Deviation	Min	Max
Glucose (mg/dL)	<160 normal >160 abnormal	Riverine	102	122.9	116.0	37.9	67.0	339.0
		Urban	123	118.7	104.0	54.3	62.0	429.0
Urea (mg/dL)	10–45 women/men	Riverine	100	34.5	32.2	15.3	13.9	115.7
		Urban	131	28.3	28.0	8.7	10.0	77.0
Creatinine (mg/dL)	0.51–1.10—women	Riverine	100	0.9	0.9	0.4	0.2	4.4
	0.7–1.20—men	Urban	131	0.9	0.8	0.3	0.3	2.3
AST (U/L)	10–37—women	Riverine	100	23.0	23.0	9.9	3.0	59.0
	11–39—men	Urban	131	33.6	31.0	22.1	12.0	232.0
ALT (U/L)	10–47—women	Riverine	100	19.8	18.0	12.0	2.0	53.0
	11–45—men	Urban	131	29.1	24.0	20.6	11.0	143.0

AST, alanine aminotransferase; ALT, aspartate aminotransferase.

**Table 4:** Descriptive analyses of the health indicators, according to residence location (riverine vs. urban), Brazilian Amazon basin, 2015–2019.

Health Indicators		Urban Area		Riverine Area		Total
		*n*	%	*n*	%	
Glucose (*n* = 225)	Normal	108	54.0	92	46.0	200
	Abnormal	15	60.0	10	40.0	25
Urea	Normal	128	60.4	84	39.6	212
	Abnormal	3	15.8	16	84.2	19
Creatinine	Normal	130	57.8	95	42.2	225
	Abnormal	1	16.7	5	83.3	6
AST	Normal	109	52.9	97	47.1	206
	Abnormal	22	88.0	3	12.0	25
ALT	Normal	114	53.5	99	46.5	213
	Abnormal	17	94.4	1	5.6	18
Blood pressure	Normal	103	39.5	158	60.5	261
	Abnormal	22	52.4	20	47.6	42
BMI	Underweight	3	25.0	9	75.0	12
	Normal weight	33	26.0	94	74.0	127
	Overweight	39	42.4	53	57.6	92
	Obese	50	65.8	26	34.2	76

## Data Availability

https://repositorio.ufopa.edu.br/jspui/handle/123456789/481, accessed on 4 January 2022.
